# Where are the polyps? Molecular identification, distribution and population differentiation of *Aurelia aurita* jellyfish polyps in the southern North Sea area

**DOI:** 10.1007/s00227-016-2945-4

**Published:** 2016-07-18

**Authors:** Lodewijk van Walraven, Floor Driessen, Judith van Bleijswijk, Anneke Bol, Pieternella C. Luttikhuizen, Joop W. P. Coolen, Oscar G. Bos, Adriaan Gittenberger, Niels Schrieken, Victor T. Langenberg, Henk W. van der Veer

**Affiliations:** 1 Department of Coastal Systems and Utrecht University, NIOZ Royal Netherlands Institute for Sea Research, PO Box 59, 1790 AB Den Burg, Texel, The Netherlands; 2 Department of Marine Microbiology, NIOZ Royal Netherlands Institute for Sea Research, PO Box 59, 1790 AB Den Burg, Texel, The Netherlands; 3 Department of Ecosystems, IMARES Wageningen UR – Institute for Marine Resource and Ecosystem Studies, PO Box 167, 1790 AD Den Burg, The Netherlands; 4 Chair group Aquatic Ecology and Water Quality Management, Wageningen UR, Droevendaalsesteeg 3a, 6708 PD Wageningen, The Netherlands; 5 GiMaRIS, J.H. Oortweg 21, 2333 CH Leiden, The Netherlands; 6 Institute of Biology Leiden (IBL), Leiden University, P.O. Box 9516, 2300 RA Leiden, The Netherlands; 7 Department of Marine Zoology, Naturalis Biodiversity Center, P.O. Box 9517, 2300 RA Leiden, The Netherlands; 8 BiOrganized, Grenadiersweg 8, 3902 JC Veenendaal, The Netherlands; 9 ANEMOON Foundation, P.O. Box 29, 2120 AA Bennebroek, The Netherlands; 10 DELTARES, P.O. Box 177, 2600 MH Delft, The Netherlands

## Abstract

For many species of metagenic jellyfish the location of the benthic polyps is unknown. To gain insight in the distribution, species composition and population structure of scyphozoan jellyfish polyps in the southern North Sea area, polyp samples were collected from natural and artificial substrates (settling plates, marina floats and wrecks) at ten inshore locations in the Netherlands, seven offshore locations in the North Sea and in the Gullmar Fjord in Sweden. Polyps were identified to species level by sequencing both a fragment of 18S rDNA and a fragment of mitochondrial COI, and comparing these sequences to reference sequences available in GenBank and to newly obtained sequences from medusae collected in the area. All polyps sequenced did belong to *Aurelia aurita*. For this species, molecular diversity in mitochondrial COI was high, with 50 haplotypes among 183 polyps. Population differentiation was detected between the Dogger Bank and other—more coastal—locations, indicating extremely low connectivity. No significant differences were found between coastal samples. The location of polyps of *Cyanea capillata*, *Cyanea lamarckii*, *Chrysaora hysoscella* and *Rhizostoma octopus* in the study area remains unresolved.

**Electronic supplementary material:**

The online version of this article (doi:10.1007/s00227-016-2945-4) contains supplementary material, which is available to authorized users.

## Introduction

A variety of anthropogenic influences is suggested to contribute to increased gelatinous zooplankton blooms, such as climate change, overfishing, depletion of predators and increased habitat availability due to coastal and offshore engineering (Richardson et al. 2009; Purcell 2012; Duarte et al. 2012; Lucas et al. 2012). Particularly, the increasing availability of artificial substrates can contribute to an increase in jellyfish blooms for species with a benthic life stage (Duarte et al. 2012). For example, the introduction of a single 48 × 6 m pier caused an estimated 4.3-fold increase in the number of immature jellyfish (ephyrae) exported from a fishing port on the Inland Sea of Japan (Makabe et al. 2014).

Many scyphozoan jellyfish species have a life cycle consisting of a sessile polyp stage and a free-swimming medusae stage. The male medusa releases sperm through its mouth into the water column. Fertilization occurs in the female or in the water column (Schiariti et al. 2012 and references therein). The fertilized egg develops into a free-swimming planula larva. The free-swimming planula larvae settle on hard substrate and metamorphose into sessile polyps called scyphistomae. Scyphistomae are a few millimeters in length (Holst and Jarms 2007), inconspicuous and typically inhabit shaded environments often underneath horizontal surfaces of rocks and shells (Pitt 2000). They settle on a wide range of artificial substrates such as breakwaters, marina pontoons, plastic waste and aluminum cans (Holst and Jarms 2007; Purcell et al. 2009; Duarte et al. 2012). Polyps are most abundant in the early stages of colonization of substrates (Lindeyer and Gittenberger 2011; Makabe et al. 2014).

Polyps of most species propagate asexually. A polyp can live for several years (Arai 1997). The transition from polyp to medusae is also a way of asexual reproduction; immature medusae (ephyrae) are released into the water column by strobilation. One polyp can produce as many as 40 ephyrae during each strobilation event (Lucas 2001). Asexual reproduction and the perennial duration of the polyp stage can result in apparently unregular and unpredictable patterns in abundance of medusae (Boero et al. 2008). Due to the small body sizes and cryptic lifestyle, these sessile stages of many jellyfish species are often unnoticed and their location unknown. Because polyps are the source of metagenic scyphozoan blooms (Arai 1997), knowledge of their distribution is key to understanding and predicting the response of scyphozoan populations to factors such as climate change (Mills 2001) and the increasing availability of artificial substrate (Duarte et al. 2012).

In areas such as the southern North Sea where natural hard substrate is absent or scarce (ICES 2016) artificial structures can be seen as “oases of marine biodiversity” because they offer hard substrate where normally only soft bottoms occur (Lengkeek et al. 2013a; Schrieken et al. 2013). With approximately 600 oil and gas installations, 2584 km^2^ wind farms and 27,000 wrecks present in the North Sea area, the availability of artificial substrates is significant (ICES 2016; Coolen et al. 2015b). Similarly, in nearshore areas, structures such as marinas offer additional settlement substrates. In the southern North Sea and bordering Dutch coastal waters, scyphozoan polyps have been found on several types of artificial structures in several locations. Lindeyer and Gittenberger (2011) found scyphistomae on PVC settling plates, suspended at 1 m depth in marinas and ports in various locations in the Eastern Scheldt and Lake Grevelingen. Polyps have also been found on other hard substrates in the same areas (De Kluijver and Leewis 1994; Gmelig Meyling et al. 2013). In the North Sea polyps are found on artificial reefs (van Moorsel 1993), on various shipwrecks (Waardenburg 1987; van Moorsel et al. 1991; Leewis and Waardenburg 1991; van Moorsel and Waardenburg 1992; Hiscock et al. 2010; Lengkeek et al. 2013b), oil platforms (Guerin 2009) and wind farm foundations (Vanagt and Faasse 2014). In most of these studies the polyps are assumed to belong to *Aurelia aurita.*

Medusae of five species of scyphomedusae are commonly found in most Dutch coastal waters: the moon jellyfish *Aurelia* sp., the compass jellyfish *Chrysaora hysoscella*, the lion’s mane jellyfish *Cyanea capillata*, the blue jellyfish *Cyanea lamarckii* and the barrel jellyfish *Rhizostoma octopus*. These are found in the North Sea (van der Baan 1980; Hay et al. 1990; Barz and Hirche 2007), Eastern Scheldt estuary (Bakker 1994) and Wadden Sea (van der Veer 1985; van Walraven et al. 2015). In the saline lake Grevelingen all species except *C. capillata* are found (Gmelig Meyling et al. 2013). Except for *Rhizostoma octopus*, these species have a metagenic life cycle where fertilization occurs in the female. Embryonic development takes place inside specific brood pouches in the oral arms in the *Cyanea* species and in *Aurelia*. In *Chrysaora hysoscella* planulae develop inside the gonads and in *Rhizostoma octopus* planulae develop externally (Russell 1970, Holst and Jarms 2007).

Experimental work has shown that the polyps of these five scyphozoan species can not be identified based on morphological features alone (Holst 2012b). None of the studies mentioned above used molecular methods to identify the polyps found, nor identified them by inducing them to strobilate and identifying the ephyrae, so it is possible that species other than *Aurelia aurita* were present. Therefore, the main goal of this study is to identify to species level scyphozoan polyps found in these locations using a slowly evolving marker (nuclear 18S rDNA) and a fast-evolving marker (mitochondrial COI) for molecular species identification.

Population subdivision is a typical find in population genetic studies of jellyfish (e.g., Dawson 2005; Ramšak et al. 2012; Lee et al. 2013). Connectivity between areas tens to hundreds of kilometers apart may be extremely low (Dawson et al. 2015), but apparent panmixis up to large geographic scales has also been observed (e.g., Stopar et al. 2010; Miller et al. 2012; Dong et al. 2015). Genetically different populations of the same species can exhibit differences in factors such as the timing and magnitude of medusae blooms (Dawson et al. 2015). Knowledge of the genetic structure of jellyfish population can thus be important in predicting when and where scyphozoan jellyfish blooms occur.

To date, studies on population structure of metagenic scyphozoa have sampled the mobile medusae, rather than the sessile polyps. Medusae can disperse over long distances during their life, while polyps are typically fixed. The second goal of this study was to investigate whether population differentiation exists in scyphozoan polyps in the southern North Sea area. When there is population differentiation between polyp populations in the area, it could be possible, for example, that the phenotypical response to changing environmental conditions could differ for polyps of the same species in different areas.

## Methods

### Specimen collection

**Fig. 1:**
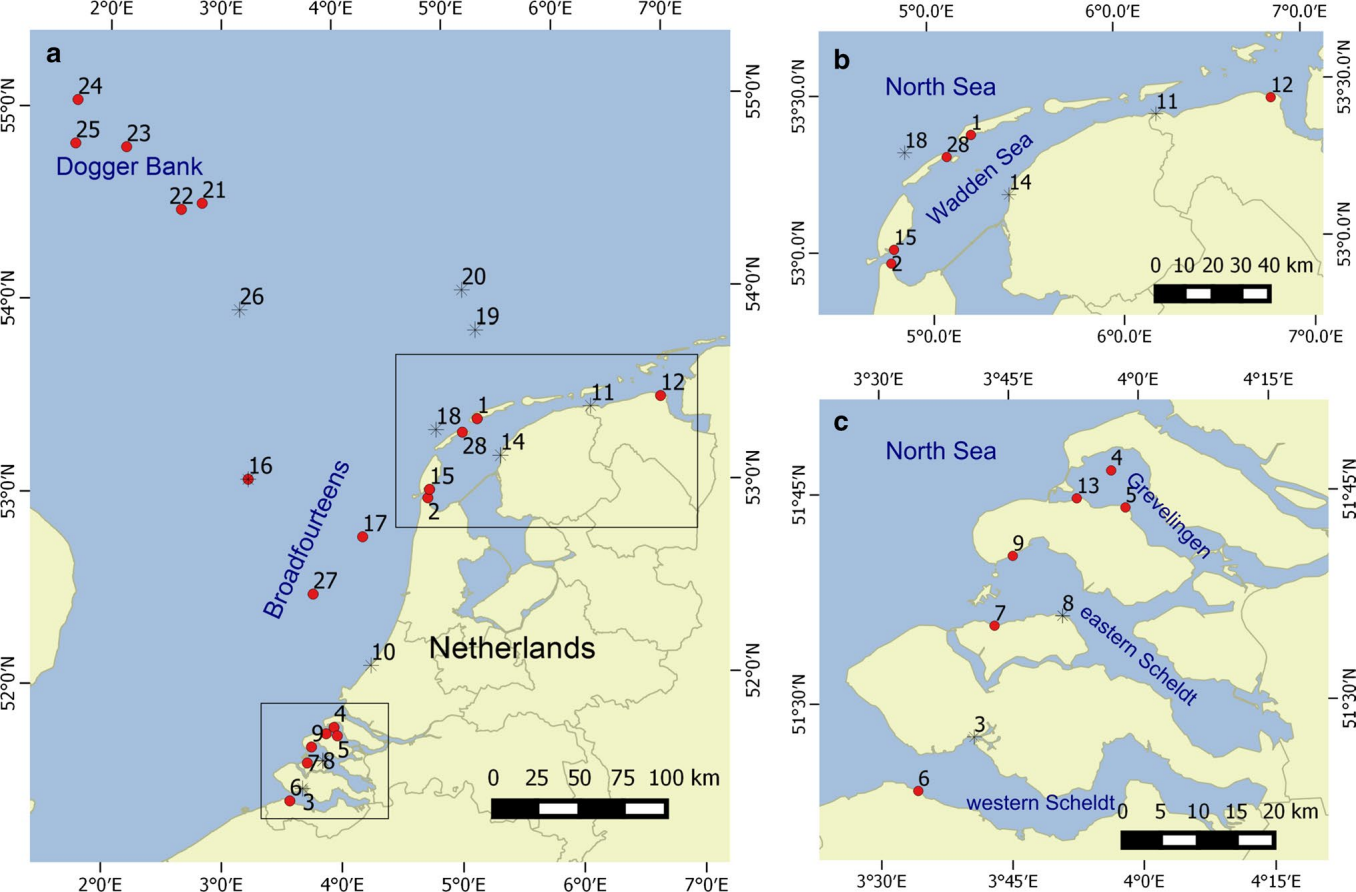
Overview of sampled locations. Polyps were found on locations indicated with *red* filled circles and not found on locations indicated with *stars*. Numbers correspond to the numbers in Table 1. **a** Whole studied area, **b** Wadden Sea, **c** Zeeland

Medusae of the five dominant species present in the area were collected from net tows and beaches at several locations in the southern North Sea area (Table 1; Fig. 1). As reference material for species identification using molecular markers, a piece of bell margin was clipped and stored in 2-ml Eppendorf cups filled with 96 % EtOH. Polyps were collected in various ways: from artificial setting plates, from floats in Dutch ports and marinas, and by scuba diving from hard underwater substrates.

**Table 1:** Overview of sampled locations

nr	Location type	Place	Area	*n*	*d* (m)	Polyps found	Coll. type	Date	Deg. N	Deg. E
1	Marina	West-Terschelling	western Wadden Sea	3	1	Yes	FFloat	05/07/2010	53.3651	5.2216
2	Marina	Den Helder	western Wadden Sea	9	1	Yes	SETL	14/12/2012	52.9616	4.7804
3	Harbor	Vlissingen	western Scheldt, Zeeland		1	No	SETL	20/12/2012	51.4598	3.6782
4	Harbor	Hompelvoet	Grevelingen, Zeeland	10	1	Yes	SETL	18/03/2013	51.7761	3.9464
5	Harbor	Bommenede	Grevelingen, Zeeland	10	1	Yes	SETL	18/03/2013	51.7317	3.9729
6	Marina	Breskens	western Scheldt, Zeeland	15	1	Yes	SETL	09/04/2013	51.3958	3.5704
7	Marina	Kamperland	eastern Scheldt, Zeeland	12	1	Yes	Float	10/04/2013	51.5923	3.719
8	Marina	Colijnsplaat	eastern Scheldt, Zeeland		1	No	Float	10/04/2013	51.6033	3.8496
8	Marina	Colijnsplaat	eastern Scheldt, Zeeland		1	No	SETL	10/04/2013	51.6033	3.8496
9	Marina	Burghsluis	eastern Scheldt, Zeeland	4	1	Yes	Float	10/04/2013	51.6755	3.7553
10	Marina	Scheveningen	central North Sea		1	No	Float	29/04/2013	52.096	4.266
11	Marina	Lauwersoog	western Wadden Sea		1	No	Float	01/05/2013	53.4099	6.2113
12	Harbor	Eemsmond	eastern Wadden Sea	7	1	Yes	Float	01/05/2013	53.4448	6.8246
13	Oyster reef	Den Osse	Grevelingen, Zeeland	2	20	Yes	SCUBA	05/05/2013	51.7434	3.8796
14	Harbor	Harlingen	western Wadden Sea		1	No	SETL	11/05/2013	53.1707	5.4133
15	Harbor	t Horntje	western Wadden Sea	3	1	Yes	SCUBA	20/05/2013	53.0056	4.7964
16	Wreck	Russian submarine	Broad Fourteens, North Sea	4	33	Yes	SCUBA	03/07/2013	53.0718	3.2326
17	Wreck	Vinca Gorthon	Broad Fourteens, North Sea	10	20	Yes	SCUBA	05/09/2014	52.7662	4.2128
18	Wreck	Vittorio Z	Frisian Front, North Sea		14.4	No	SCUBA	06/09/2014	53.3142	4.8662
19	Wreck	Unknown	Frisian Front, North Sea		33.7	No	SCUBA	06/09/2014	53.8263	5.2267
20	Wreck	Healdton	Frisian Front, North Sea		37.4	No	SCUBA	07/09/2014	54.0342	5.1198
21	Wreck	wreck nr. 59,695	Dogger Bank, North Sea	5	33.3	Yes	SCUBA	09/09/2014	54.5035	2.8293
22	Wreck	Ocean Prince	Dogger Bank, North Sea	14	29.4	Yes	SCUBA	09/09/2014	54.4712	2.644
23	Wreck	wreck nr. 70,502	Dogger Bank, North Sea	10	27	Yes	SCUBA	10/09/2014	54.7952	2.1482
24	Wreck	wreck nr. 70,500	Dogger Bank, North Sea	4	25	Yes	SCUBA	10/09/2014	55.037	1.7027
25	Wreck	wreck nr. 70,501	Dogger Bank, North Sea	8	32.8	Yes	SCUBA	11/09/2014	54.8108	1.6898
26	Wreck	Britta	Cleaver Bank, North Sea		38.5	No	SCUBA	12/09/2014	53.9488	3.1618
16	Wreck	Russian submarine	Broad Fourteens, North Sea		33	No	SCUBA	12/09/2014	53.0718	3.2326
27	Wreck	wreck “vaderdag”	Broad Fourteens, North Sea	10	31	Yes	SCUBA	13/09/2014	52.4713	3.7827
28	Marina	Oost-Vlieland	western Wadden Sea	10	1	Yes	SCUBA	26/09/2014	53.2969	5.089
15	Harbor	t Horntje	western Wadden Sea	8	3	Yes	Float	26/09/2014	53.0056	4.7964
1	Marina	West-Terschelling	western Wadden Sea	8	1	Yes	Float	26/09/2014	53.3651	5.2216
29	Marina	Kristineberg	Gullmar Fjord, Skagerrak	17	1	Yes	Snorkel	30/09/2013	58.2499	11.4465

*n* number of sequences obtained, *d* depth in meters

**Fig. 2:**
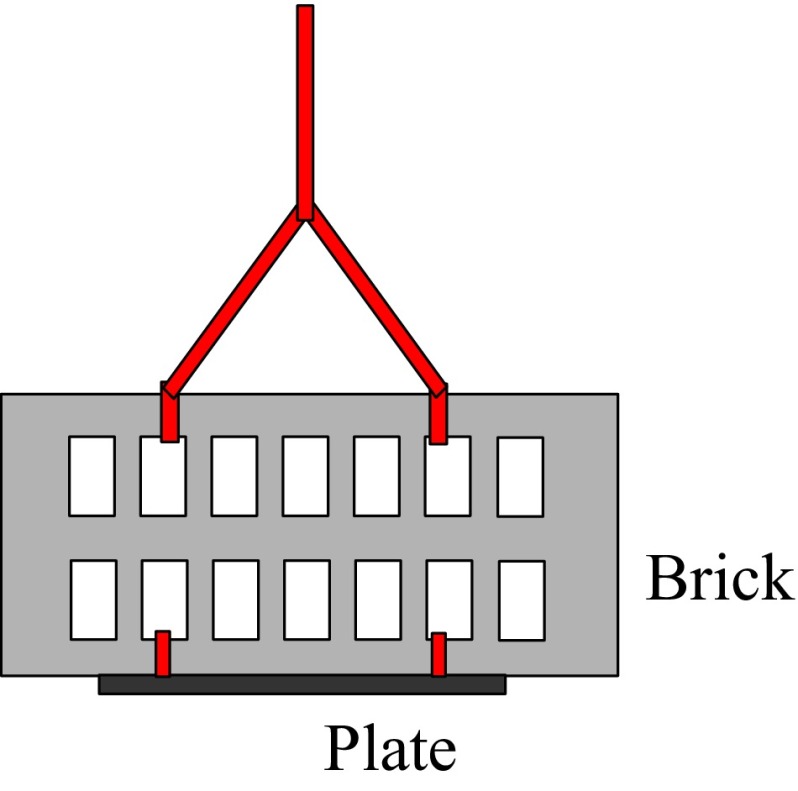
The SETL plate design. The PVC plate is attached to the brick with zip ties. The brick is 21.2 cm long, 9.7 cm high and 5.2 cm wide

Settling plates were deployed at 1 m depth in various Dutch marinas and ports as part of an ongoing program aimed at monitoring the presence of invasive species (the SETL program of the ANEMOON foundation). The plates consisted of a 14 × 14 cm 0.5-cm thick gray PVC of which the bottom side was sanded to create a rough surface. Plates were attached to a standard brick (Fig. 2) and deployed at a standard depth of 1 m and are periodically checked as described in Lindeyer and Gittenberger (2011). Between December 2012 and May 2013 settling plates were removed from the water and, submerged, checked for the presence of scyphozoan polyps by eye on eight different locations. If present, a minimum of two polyps per plate were removed with tweezers and stored in 2-ml Eppendorf cups in seawater. Using a binocular microscope, the polyps were cleaned of debris and remains of the substrate, after which they were stored individually in 2-ml Eppendorf cups filled with 96 % EtOH.

Checking epifauna for the presence of polyps and subsequent scraping epifauna from the bottom of floats was done in nine marinas at a minimum of three locations per site between March 2013 and September 2014. On site, the epifauna was inspected while submerged for the presence of polyps, which if present were collected the same way as described for the settling plates. Three polyps collected in 2010 in the marina of West-Terschelling by Floris Bennema were also included in the analysis. This location was resampled in September 2014. Additional polyps were collected from the underside of the dock of the Lovén Centre in Kristineberg, located at the Gullmar Fjord in western Sweden during a visit in September 2013.

Polyps were collected by recreational scuba divers at one site in lake Grevelingen and two sites in the western Wadden Sea. Additionally, polyps were collected by scuba diving on North Sea wrecks during two expeditions organized by Stichting Duik de Noordzee Schoon and the World Wildlife Fund on the vessel Cdt. Fourcault from June 28 to July 6, 2013, and September 5–13, 2014. These expeditions aimed at investigating the biodiversity of North Sea shipwrecks and have been organized since 2011. Applied methods and results are published in Schrieken et al. (2013), Lengkeek et al. (2013a) and Coolen et al. (2015a). Dives were made by following a 45-m-long reel line laid out by the first dive pair. Wreck parts in close (max 5 m) proximity to the line were inspected for the presence of scyphozoan polyps. The wrecks were not entered. When polyps were found, they were collected by scraping the polyps and their substrate using a filling knife with one hand, collecting the scrapings in a 1-l Kautex jar held below the patch with the other hand. In hard-to-reach areas where only one hand could reach the polyp patch, polyps were collected by scraping slowly over the patch with the edge of a 50-ml centrifuge tube. Polyps were collected from as many different patches as possible. On board the polyps were cleaned and stored as described before. A minimum of 5 polyps per patch were collected.

### DNA extraction and amplification

DNA was extracted from medusae and two to five (depending on availability) individual polyps per patch using the Power Soil DNA Isolation Kit (MO BIO Laboratories Inc.) following the manufacturers’ protocol. DNA was eluted from silica columns in 50 μl of buffer, quantified with NanoDrop and run on 1 % agarose gels to verify the quality of the extract. Species identification was performed in two steps. For a first identification, diagnostic fragments of 650 bp of the V4 and V5 regions in the 18S rDNA gene were amplified from 2 μl of DNA extract in a 50-μl PCR using the primers EUK_F_566 and EUK_R_1200 according to Hadziavdic et al. (2014). The reaction mix contained 1 × buffer, 200 μM dNTPs, 0.5 μM forward and reverse primers, 0.8 mg/ml BSA and 1 u Biotherm Plus Polymerase. PCR amplification started with 2 min at 95 °C followed by 35 cycles of 45 s at 95 °C, 60 s at 60 °C, 60 s at 72 °C and a final step of 7 min at 72 °C. Positive PCR controls (non-scyphozoan metazoa) and negative PCR controls were processed along. To test whether the extraction and PCR protocols worked correctly on scyphozoan polyps, the method was tested using three polyps of *Cyanea lamarckii* and three polyps of *C. capillata* provided from the cultures of Senckenberg am Meer by Dr. Sabine Holst. PCR products were sequenced by BaseClear (Leiden) in a single run with forward primer EUK_F_566.

Subsequently, based on the genus to which the polyps were assigned, a fragment of fast-evolving mitochondrial COI was amplified using a primer pair suitable for that genus, as different authors have used different primer pairs to amplify mitochondrial COI in the genera considered (Ramšak et al. 2012; Lee et al. 2013; Holst and Laakmann 2014).

We used newly designed primers for analyses of intra-specific variation of *Aurelia aurita* polyps, based on GenBank data for the mitochondrial cytochrome c oxidase subunit I gene (COI) of *Aurelia aurita*: JQ623914, KC311384, KC311385, AY428838, AY903093–AY903095, AY903117, AY903208–AY903212, EF010537, DQ904436–DQ904439, FJ858784, EF010537, AY902911, AY902924; *Cyanea capillata*: AY902911, AY902924 and *Rhizostoma pulmo* (as *R. octopus* data were not available at the time): HQ902114–HQ902122, HQ904432–HQ904435, HQ999568–GQ999571:

Forward: ScyCOIf (5′-CTATACTTAATATTTGGTGCYTTTTC-3′).

Reverse: ScyCOIr (5′-AAATGTTGGAATARTATTGGRTCTCCT-3′).

PCR amplification started with 5 min at 94 °C followed by 40 cycles of 30 s at 94 °C, 45 s at 55 °C, 45 s at 72 °C and a final step of 7 min at 72 °C. Subsamples (5 μl) of all PCR products were loaded on 2 % agarose gels along with a size marker (SmartLadder SF) and stained with EtBr. The presence of bands was scored visually. Remaining volume (45 μl) of respective PCR products was submitted to BaseClear (Leiden) for purification and sequencing in two runs with primers ScyCOIf and ScyCOIr for intra-specific analyses of *Aurelia aurita*.

### Data analysis

For species identification of the polyps, reads of the 18S rRNA gene were trimmed to 564-bp high-quality fragments and, together with sequences from relevant Scyphozoa from GenBank, aligned to the Silva 119 reference database (Quast et al. 2013) using ARB (Ludwig et al. 2004). These sequences were added to the Silva guide tree using ARB Parsimony with positional variability settings specific for eukaryotes. Subsequently, from a subset of Scyphozoa sequences (1564–1684 bp) a small Maximum Likelyhood (ARB-RaxML) tree was built to which the sequences from this study (563–574 bp) were added using ARB Parsimony. Polyp species were identified based on the position of their sequences in the trees. For all polyps that were identified as belonging to the genus *Aurelia* based on their 18S rRNA sequence, forward and reverse sequences of COI were assembled and the consensus sequences were trimmed to 473-bp fragments. These were imported into Arlequin (Excoffier and Lischer 2010) for standard diversity analyses, calculating pairwise F_st_ and analysis of molecular variance. A haplotype network was computed in R using the R package Pegas (Paradis 2010). The genus *Aurelia* contains several cryptic species (Dawson et al. 2005), and GenBank sequences of each species published in Dawson et al. (2005) were added to a COI tree to identify the polyps to species level.

Jellyfish polyps can reproduce asexually in different ways which means that polyps collected close to each other, for example in one patch, are likely to be clones. Including all polyps collected from a patch in an analysis of molecular variance would thus violate the assumption that individuals are sampled randomly from a population. For this reason, one polyp sequence from each polyp patch was randomly selected to be included in the analysis. Polyps were treated as separate individuals if they were on a different SETL plate, a different host organism, or clearly separated by distance, for example on a different wreck part. Sequences of this study are available from GenBank (KT962253–KT962259 for 18SrDNA and KP728285–KP728377 for COI).

## Results

### Polyp distribution

Jellyfish polyps were found inshore at four out of seven SETL settling plate locations and at seven out of nine locations where marine floats were sampled (Fig. 1b, c). Offshore in the Dutch and Great British Exclusive Economic Zone (EEZ) polyps were found at eight out of eleven dive locations; five locations on Dogger Bank and three in the “Broad Fourteens” area (Fig. 1a). Five of the wrecks where polyps were found were steamers, one a submarine, one a sunken oil rig (Ocean Prince) and one, the most recent wreck, the merchant motor vessel Vinca Gorthon sunk in 1988. Depths of these wrecks ranged from 20 to 34 m. On the wrecks the polyps were generally found on parts that were sheltered from the current such as beam joints and the insides of pipes and boiler parts.

Polyps were found on a wide range of abiotic and biotic substrates (Table 2). Mostly, polyps were attached to the PVC of settling plates and to the oxidized metal surface of the wrecks. Polyps were also found on wood, granite pebbles, glass and synthetic rubber. A wide range of organisms was host to polyp patches: barnacles (both empty and alive), three species of bivalves, four species of tunicates, a sponge and a bryozoan.

**Table 2:** Overview of substrates on which polyps were found with site numbers corresponding to the site number in Table 1

	Substrate	Site nr
Abiotic		
	PVC	2, 4, 5, 6
	Synthetic rubber	9
	Iron oxide (rust)	16, 17, 23, 24, 25, 27
	Wood	29
	Granite	29
	Glass	29
Biotic		
	*Crassostrea gigas*	7, 15, 28
	*Mytilus edulis*	1, 9, 12, 15, 28, 29
	*Pododesmus squamulatus*	27
	*Semibalanus/balanus* sp.	1, 13, 15, 28
	Encr. Bryozoan	27
	*Aplidium glabrum*	1
	*Ascidiella aspersa*	22
	*Ciona intestinalis*	7
	*Styela clava*	15, 28
	*Protosuberites* sp.	1

### Molecular identification

**Fig. 3:**
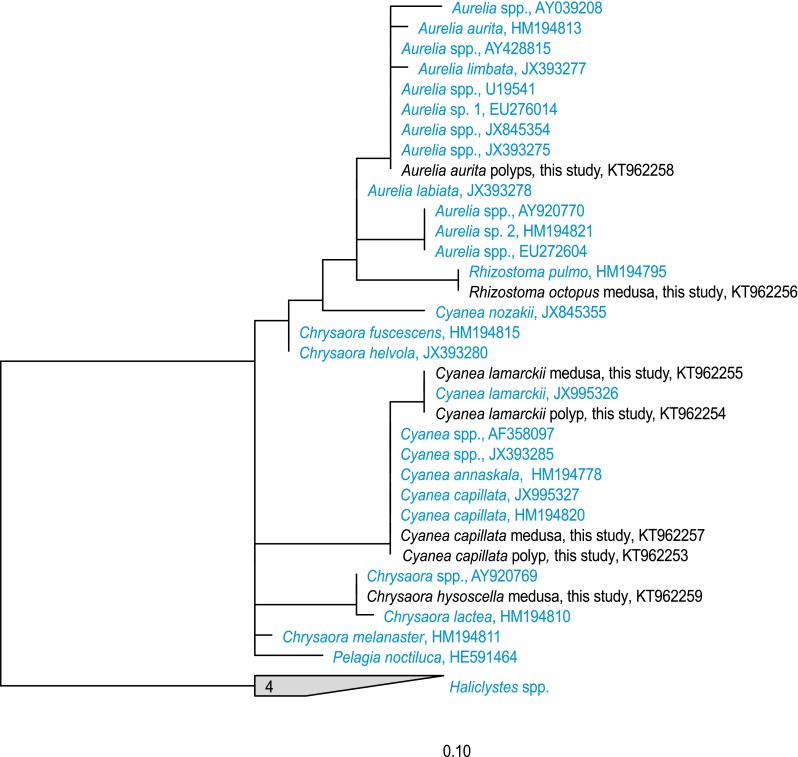
Maximum Likelyhood (ARB-RaxML) tree of partial 18S rRNA genes of Scyphozoa with sequences from this study in blue added using ARB Parsimony (Ludwig et al. 2004)

Nuclear 18S rDNA sequences of medusae and polyps used as reference material grouped with sequences of their respective genera in GenBank (Fig. 3). The fragment analyzed (563–574 bp) did not allow discrimination between *Rhizostoma pulmo* and *Rhizostoma octopus* and, e.g., *Cyanea capillata* and *Cyanea annaskala*. 18S rDNA sequencing of polyps had a success rate of 85 % and worked for 183 samples. All sequences obtained from polyps were identical and matched to sequences of *Aurelia* sp. available in GenBank (Fig. 3). Polyps of other genera were not detected. Subsequent analyses of the cytochrome c oxidase gene using the newly designed ScyCO primers were successful for 93 % of the *Aurelia* polyps. All polyps belonged to the species *Aurelia aurita* (Figure S1). Variation within the 473-bp mitochondrial COI fragment was high with 52 variable sites and 50 different haplotypes with pairwise K80 distances ranging from 0 to 3 %.

### Genetic structure

After randomly sampling one sequence per polyp patch to avoid including clonal genotypes, 63 sequences remained which were used in further analyses (Table 3). Haplotype richness was high (*h* = 0.967 ± 0.011 on average), but differentiation among haplotypes was modest (*π* = 0.01,080 ± 0.0007). Pairwise F_ST_ values indicated significant population differentiation between the Dogger Bank sample and other samples: Broad Fourteens, Skagerrak, Zeeland and Wadden Sea (Table 4); after Bonferroni correction, only the contrasts Dogger Bank versus Broad Fourteens, Wadden Sea and Zeeland remained significant. The largest level of genetic differentiation was found between the Broad Fourteens and Dogger Bank areas (*F*_ST_ = 0.489) and the smallest difference between the Wadden Sea and Zeeland (*F*_ST_ = −0.013). Based on the pairwise *F*_ST_ outcomes, two analyses of molecular variance (AMOVA) were carried out, both with sequence divergence taken into account. The first consisted of sites nested within two areas (outer area with Dogger Bank only versus coastal area with the other four samples). It showed a significant difference between outer and coastal areas but not among sites within areas (10.000 permutations, *F*_ST_ = 0.157 (*p* = 0.01), *F*_SC_ = −0.006 (*p* = 0.271), *F*_CT_ = 0.162 (*p* = 0.001)). The second AMOVA was single-level with Dogger Bank versus the other samples pooled, confirming the different status of the Dogger Bank sample (10.000 permutations, *F*_ST_ = 0.227, *p* = 0.009).

**Table 3:** Sample sizes and standard diversity indices for partial COI sequences of *Aurelia aurita* polyps sampled at five locations

Area	*N*	*N*_h_	*h* ± sd	*π* ± sd
Broad Fourteens	4	3	0.833 ± 0.222	0.00,247 ± 0.0023
Dogger Bank	10	5	0.822 ± 0.097	0.00,583 ± 0.00,381
Skagerrak	7	6	0.952 ± 0.096	0.00,785 ± 0.00,516
Wadden Sea	18	14	0.967 ± 0.03	0.0127 ± 0.0071
Zeeland	29	18	0.936 ± 0.034	0.00,976 ± 0.00,549
Total	63	38	0.967 ± 0.011	0.0108 ± 0.0007

*N* sample size, *N*_h_ number of haplotypes observed in sample, *h* haplotype diversity, *π* nucleotide diversity

**Table 4:** Pairwise F_ST_ values among samples of *Aurelia aurita* polyps in the North Sea area

	Broad Fourteens	Dogger Bank	Skagerrak	Wadden Sea
Dogger Bank	0.489***	–	–	–
Skagerrak	0.095	0.306**	–	–
Wadden Sea	0.05	0.310***	0.121	–
Zeeland	0.037	0.341***	0.096	−0.013

Bonferroni-corrected threshold value is *p* = 0.005

* *p* < 0.05; ** *p* < 0.005; *** *p* < 0.001

**Fig. 4:**
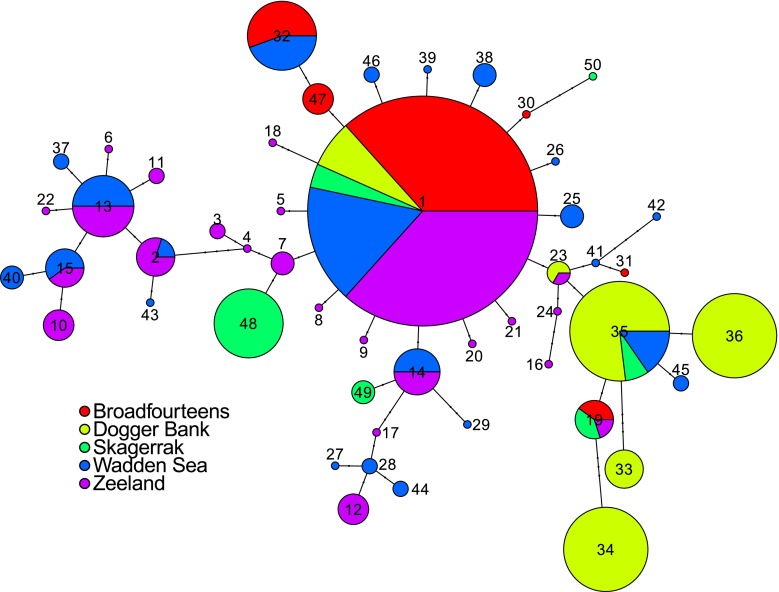
Haplotype network of the 473-bp mitochondrial COI fragments of all *Aurelia aurita* polyps sequenced in this study, computed using the R package “pegas.” *Circle* size is proportional to the frequency of the haplotype, and *circles* are *colored* based on the proportion of individuals from the five different geographic areas sampled in this study. *Lines* represent mutational steps with *black* *dots* as hypothetical intermediate haplotypes

A haplotype network was computed for the 473-bp mitochondrial COI fragments of all 183 *Aurelia aurita* polyps successfully sequenced in this study (Fig. 4) and showed that the most frequently found haplotype 1 was shared between all locations. The coastal areas Wadden Sea and Zeeland shared most haplotypes with each other. The Dogger Bank area shared the least haplotypes with other areas.

One polyp COI sequence of each haplotype/location combination was deposited in GenBank (Table S1).

## Discussion

*Aurelia aurita* polyps were found in nearshore waters on settling plates, marina floats and other artificial as well as biological substrates, and offshore on several shipwrecks and an oil rig wreck on the Dogger Bank and the Broad Fourteens. *Aurelia aurita* polyps were found in most of the marinas and ports sampled in this study, suggesting marinas are an important source of *A. aurita* ephyrae in The Netherlands. Several of the Dogger Bank locations at which polyps were found are in the Exclusive Economic Zone of the UK. In this area a total of nine gigawatt of offshore wind turbines is planned (Toke 2011). Our study shows that settlement of scyphozoan planulae occurs in the Dogger Bank area. The structures installed for the wind farm would mean an increase in available polyp habitat and likely an increased release of ephyrae from the area, as has been hypothesized for wind farms in the Baltic Sea (Janßen et al. 2013) as well.

Before the onset of industrial fisheries in the 1900s, large *Ostrea edulis* reefs were present on the Dogger Bank and in the area between the Dogger Bank and the Dutch-German coasts (Coolen et al. 2015a). These reefs are now functionally extinct in the North Sea (Beck et al. 2011), but may have provided a similar function to the benthic stages of jellyfish that is now provided by artificial structures.

### Species composition: only *Aurelia aurita*

There was sufficient variation in the 18S rDNA fragment sequenced to distinguish between the five species of metagenic scyphomedusae known to be present in the North Sea area but not enough variation to distinguish between all species at a worldwide scale (Fig. 3). As several species of *Aurelia* are cryptic and are thought to be introduced species (Dawson 2005), it is possible that these also occur in the North Sea area unnoticed. Therefore, species identification using a faster evolving fragment, mtCOI, was required and applied.

Previous work mentioning the occurrence of jellyfish polyps in Dutch coastal waters assumed that these all belonged to *Aurelia aurita* (van Moorsel et al. 1991; van Moorsel and Waardenburg 1992; van Moorsel 1993; Lindeyer and Gittenberger 2011; Gmelig Meyling et al. 2013). In the present study, all polyps were indeed assigned to *Aurelia* sp. based on 18S rDNA and *Aurelia aurita* based on COI. However, based on our data it cannot be excluded that in previous work polyps of other species were present and it is recommended that any future study on field-sampled polyps includes species identification based either on molecular identification or on the traditional method of rearing, strobilation and identification of ephyrae. Ephyrae of all North Sea species (Holst 2012a, b) as well as many other species (Straehler-Pohl and Jarms 2010; Straehler-Pohl et al. 2011) can be identified to species level.

There are several possible explanations for the fact that only *Aurelia aurita* scyphistomae were found. First, it could be an effect of substrate preference. Experimental work by Holst and Jarms (2007) and Hoover and Purcell (2009) found that all species considered in this study settle on a variety of artificial substrates including plastic (PET). In the field, polyps of *Aurelia* species have been found on artificial substrates in many areas worldwide (Duarte et al. 2012). Polyps of *Cyanea capillata* were only found once on natural substrate; on granite rock near the island of Helgoland in the German Bight (Hartlaub 1894). In the Gullmar Fjord, despite the high abundance of *C. capillata* females bearing planula, few polyps colonized ceramic settling plates while the same plates were colonized extensively by *A. aurita* (Gröndahl 1988) suggesting that there are differences in settlement preference.

Second, the different scyphozoan species could have preferences for different environmental conditions; if there are no adults in a certain area, no polyps are to be expected, either. Based on the more offshore distribution of *Cyanea lamarckii* and *C. capillata* in the North Sea, Hay et al. (1990) infer that the polyps of these species may prefer more saline and deeper waters. It was not anticipated that polyps collected in the Dogger Bank area of the central North Sea would all belong to *Aurelia aurita*, because *A. aurita* medusae are seldom numerous in this area (Hay et al. 1990), and according to Thiel (Thiel 1962) who studied *A. aurita* polyps in different locations in the Kiel Fjord, *A. aurita* polyps seem to prefer less saline waters.

Experimental observations on *Aurelia aurita*, *Cyanea lamarckii*, *C. capillata* and *Chrysaora hysoscella* indicate a high tolerance of their planulae and polyps to low salinity. Planulae of *C. capillata*, *C. lamarckii* and *C. hysoscella* settled in salinities down to 20 and polyps of *C. capillata*, *C. lamarckii* and *A. aurita* survived exposure to salinities down to 12 (Holst and Jarms 2010) indicating that they can settle and survive in the estuaries bordering the southern North Sea including the Wadden Sea. Similar results were found for water temperature. For *Rhizostoma octopus* Holst et al. (2007) found that polyps could survive and reproduce at water temperatures experienced in the German Bight. In *A. aurita*, *C. lamarckii*, *C. capillata* and *C. hysoscella* strobilation activity differed between different temperature regimes but polyps of all these species survived and strobilated at average German Bight water temperature regimes as well (Holst 2012a). Based on these observations, polyps of all species could potentially be found in the southern North Sea area.

The presence or absence of ephyrae in an area can be an indication whether polyps are present nearby. The presence of *Rhizostoma octopus* ephyrae in the Elbe estuary (Thiel 1966) suggests that the polyps of this species would occur in less saline waters. Using similar reasoning, Merck (1989) suggests that the ephyrae of *Chrysaora hysoscella* might originate from the estuaries bordering the German Bight as they were found predominantly in the outflow of the river Elbe. In 2013, ephyrae of *C. hysoscella* and *Cyanea lamarckii* were found in plankton samples taken in the Eastern Scheldt and post-ephyrae of *Rhizostoma octopus* were found in the Wadden Sea (L. van Walraven unpublished data). Van der Baan (1980) studied the seasonal patterns of ephyrae and medusae of scyphomedusae extensively based on plankton samples taken in 1961–1966 from the lightvessel “Texel” 10 nm off the island of Texel. She found post-ephyrae of *Aurelia aurita* occasionally in January but most often in April and later. Small post-ephyrae of *C. hysoscella* were much less numerous and post-ephyrae of *R. octopus* were never observed, although van der Baan (van der Baan 1980) mentions that these were often observed in the Wadden Sea. Post-ephyrae (2–10 mm diameter) of *C. lamarckii* were found from November to June in high densities.

Both medusa and polyp environmental preferences and tolerances have likely contributed to the lack of polyp species identifications other than *A. aurita* in the present study. The whereabouts of polyps of the other species in the southern North Sea region thus remains an open question.

### Intra-specific variation in Aurelia

The separate genetic status of *Aurelia aurita* polyps on the Dogger Bank confirms the observation by several other authors that populations of jellyfish may be differentiated at geographic scales of tens to hundreds of kilometers (see Dawson et al. 2015 for a review).

Variation in the COI gene of *Aurelia aurita* appears to be very high as 50 different haplotypes were found among the 183 polyps included in this study. A study that sampled medusae from southern English waters and the Irish Sea found 36 different haplotypes (Dawson et al. 2015), of which several were also found in this study. High levels of diversity are in fact to be expected in pelagic species with supposedly large population sizes, and this is indeed observed in many zooplankton taxa (see Peijnenburg and Goetze 2013 for a review).

Analysis of the 473-bp COI sequences revealed population subdivision in the study area. The four nearshore and inshore areas Wadden Sea, Zeeland estuaries, Broad Fourteens and Skagerrak did not differ significantly from each other in contrast to the Central North Sea Dogger Bank area which differed significantly from the Wadden Sea and Zeeland samples, and showed a trend toward differentiation from the Broad Fourteens and Skagerrak samples. The lack of connectivity may be a result of the prevailing hydrodynamic circulation and water currents in the North Sea (Otto et al. 1990). The Dogger Bank area mainly receives input of Atlantic water from the north, and not from the south (Turrell 1992). Two studies modeling the dispersal of *Mnemiopsis leidyi* ctenophores from coastal areas to the North Sea found that particles released near the Dutch coast were rarely transported to the Dogger Bank area and vice versa (van der Molen et al. 2015; David et al. 2015). A similar pattern of transport could explain the genetic differentiation found between Dogger Bank and coastal *A. aurita* polyps.

Genetic diversity of *Aurelia aurita* polyps in the southern North Sea area was higher than that found for medusae of *Aurelia* sp. within Australia, California and Japan (Dawson et al. 2005), within several European seas (Ramšak et al. 2012) and between the Irish Sea and southern England (Dawson et al. 2015). Dawson et al. (2015) show that genetically different populations of jellyfish of the same species can have different seasonal patterns and could respond differently to changing environmental conditions. Previous population genetic studies on scyphomedusae were based on medusae samples. The current study is the first that uses the sessile benthic stage and not the mobile free-living medusae and shows that also in polyps population differentiation on similar spatial scales is found as for medusae. It would be interesting to investigate whether the phenotypes of these different populations are also different.

Several hurdles are encountered when sampling polyps rather than medusae. The first one is the problem of contamination, either by ingested prey material or by inclusion of the biotic substrate on which the polyps are living. Occasionally (11 % of all processed polyps in our study) this resulted in complex electropherograms and mixed sequences when using universal primers such as EUK_F_566 and EUK_R_1200. The second aspect to take into account are the various modes of asexual reproduction used by the polyps, which means that multiple polyps sampled from the same patch are likely clones and can not be assumed to be independent. This reduces the amount of samples available for analysis of population structure. Using additional genetic markers such as microsatellite DNA would help to distinguish clones as well as provide higher resolution information on population connectivity (see, e.g., Meirmans and Van Tienderen 2004; Luttikhuizen et al. 2007).

### Polyps of other species: where are they located?

Our study did not elucidate a clue on the location of the polyps of *Cyanea capillata*, *C. lamarckii*, *Chrysaora hysoscella* and *Rhizostoma octopus* in the southern North Sea area although they should be present based on the occurrence of the medusae. Alternative methods could be employed to search for the origin of the medusae. When small medusae or ephyrae are found, hindcasting hydrodynamic models could be used to trace the medusae back to their origin, as shown by Dulière et al. (2014). Settling plates have been used to investigate settlement of planulae in an area (Gröndahl 1988; Janßen et al. 2013). Perhaps polyps of species other than *A. aurita* are more cryptic, i.e., not easily visible by eye, in which case bulk benthic scrapings from promising locations can be checked for the presence of polyps using next-generation sequencing techniques or species-specific primers and quantitative PCR as demonstrated in Bayha and Graham (2009), or microarrays (Ki et al. 2010). Water samples can be checked for the presence of planulae of certain species, similar to what has been done for the identification of bivalve veligers (Philippart et al. 2014). Environmental DNA sampling which has been employed in the marine environment before (Thomsen et al. 2012) could give useful information on the presence of nearby polyps when it is performed in periods when medusae are absent.

## Electronic supplementary material

Below is the link to the electronic supplementary material.

Supplementary material 1 (PDF 44 kb)

Supplementary material 2 (XLSX 14 kb)

## References

[CR1] Arai M (1997). A Functional Biology of Scyphozoa.

[CR2] Bakker C (1994). Zooplankton species composition in the Oosterschelde (SW Netherlands) before, during and after the construction of a storm surge barrier. Hydrobiologia.

[CR3] Barz K, Hirche HJ (2007). Abundance, distribution and prey composition of scyphomedusae in the southern North Sea. Mar Biol.

[CR4] Bayha K, Graham W (2009). A new Taqman© PCR-based method for the detection and identification of scyphozoan jellyfish polyps. Hydrobiologia.

[CR5] Beck MW, Brumbaugh RD, Airoldi L, Carranza A, Coen LD, Crawford C, Defeo O, Edgar GJ, Hancock B, Kay MC (2011). Oyster reefs at risk and recommendations for conservation, restoration, and management. Bioscience.

[CR6] Boero F, Bouillon J, Gravili C, Miglietta MP, Parsons T, Piraino S (2008). Gelatinous plankton: irregularities rule the world (sometimes). Mar Ecol Prog Ser.

[CR7] Coolen JW, Bos OG, Glorius S, Lengkeek W, Cuperus J, van der Weide B, Agüera A (2015). Reefs, sand and reef-like sand: a comparison of the benthic biodiversity of habitats in the Dutch Borkum Reef Grounds. J Sea Res.

[CR8] Coolen JW, Lengkeek W, Lewis G, Bos O, van Walraven L, van Dongen U (2015). First record of *Caryophyllia smithii* in the central southern North Sea: artificial reefs affect range extensions of sessile benthic species. Mar Biodivers Rec.

[CR9] David C, Vaz S, Loots C, Antajan E, van der Molen J, Travers-Trolet M (2015). Understanding winter distribution and transport pathways of the invasive ctenophore Mnemiopsis leidyi in the North Sea: coupling habitat and dispersal modelling approaches. Biol Invasions.

[CR10] Dawson MN (2005). Incipient speciation of *Catostylus mosaicus* (Scyphozoa, Rhizostomeae, Catostylidae), comparative phylogeography and biogeography in south-east Australia. J Biogeogr.

[CR11] Dawson M, Gupta A, England M (2005). Coupled biophysical global ocean model and molecular genetic analyses identify multiple introductions of cryptogenic species. Proc Natl Acad Sci USA.

[CR12] Dawson MN, Cieciel K, Decker MB, Hays GC, Lucas CH, Pitt KA (2015). Population-level perspectives on global change: genetic and demographic analyses indicate various scales, timing, and causes of scyphozoan jellyfish blooms. Biol Invasions.

[CR13] De Kluijver M, Leewis R (1994). Changes in the sublittoral hard substrate communities in the Ooster-schelde estuary (SW Netherlands), caused by changes in the environmental parameters. Hydrobiologia.

[CR14] Dong Z, Liu Z, Liu D (2015). Genetic characterization of the scyphozoan jellyfish *Aurelia* spp. in Chinese coastal waters using mitochondrial markers. Biochem Syst Ecol.

[CR15] Duarte CM, Pitt KA, Lucas CH, Purcell JE, Si Uye, Robinson K, Brotz L, Decker MB, Sutherland KR, Malej A (2012). Is global ocean sprawl a cause of jellyfish blooms?. Front Ecol Environ.

[CR16] Dulière V, Kerckhof F, Lacroix G (2014). Where is my jelly?. De Strandvlo.

[CR17] Excoffier L, Lischer HE (2010). Arlequin suite ver 3.5: a new series of programs to perform population genetics analyses under Linux and Windows. Mol Ecol Resour.

[CR18] Gmelig Meyling A, Lente I, Schrieken N, Gittenberger A, de Bruyne R (2013). Het Duiken Gebruiken 3. Gegevensanalyse van het Monitoringproject Onderwater Oever (MOO), Fauna-onderzoek met sportduikers in Oosterschelde en Grevelingenmeer. Periode, t/m 2012. Tech. rep..

[CR19] Gröndahl F (1988). A comparative ecological study on the scyphozoans *Aurelia aurita*, *Cyanea capillata* and *C. lamarckii* in the Gullmar Fjord, western Sweden, 1982–1986. Mar Biol.

[CR20] Guerin AJ (2009) Marine communities of North Sea offshore platforms, and the use of stable isotopes to explore artificial reef food webs. Ph.D. thesis, University of Southampton

[CR21] Hadziavdic K, Lekang K, Lanzen A, Jonassen I, Thompson EM, Troedsson C (2014). Characterization of the 18S rRNA gene for designing universal eukaryote specific primers. PLoS ONE.

[CR22] Hartlaub C (1894). Die Coelenteraten Helgolands.

[CR23] Hay SJ, Hislop JRG, Shanks AM (1990). North-Sea Scyphomedusae -Summer distribution, estimated biomass and significance particularly for 0-group Gadoid fish. Neth J Sea Res.

[CR24] Hiscock K, Sharrock S, Highfield J, Snelling D (2010). Colonization of an artificial reef in south-west England—ex-HMS “Scylla”. J Mar Biol Ass UK.

[CR25] Holst S (2012). Effects of climate warming on strobilation and ephyra production of North Sea scyphozoan jellyfish. Hydrobiologia.

[CR26] Holst S (2012). Morphology and development of benthic and pelagic life stages of North Sea jellyfish (Scyphozoa, Cnidaria) with special emphasis on the identification of ephyra stages. Mar Biol.

[CR27] Holst S, Jarms G (2007). Substrate choice and settlement preferences of planula larvae of five Scyphozoa (Cnidaria) from German Bight, North Sea. Mar Biol.

[CR28] Holst S, Jarms G (2010). Effects of low salinity on settlement and strobilation of Scyphozoa (Cnidaria): is the lion’s mane *Cyanea capillata* (L.) able to reproduce in the brackish Baltic Sea?. Hydrobiologia.

[CR29] Holst S, Laakmann S (2014). Morphological and molecular discrimination of two closely related jellyfish species, *Cyanea capillata* and *C. lamarckii* (Cnidaria, Scyphozoa), from the northeast Atlantic. J Plankton Res.

[CR30] Holst S, Sotje I, Tiemann H, Jarms G (2007). Life cycle of the rhizostome jellyfish *Rhizostoma octopus* (L.) (Scyphozoa, Rhizostomeae), with studies on cnidocysts and statoliths. Mar Biol.

[CR31] Hoover RA, Purcell JE (2009). Substrate preferences of scyphozoan *Aurelia labiata* polyps among common dock-building materials. Hydrobiologia.

[CR32] ICES (2016). ICES ecosystem overviews—greater North Sea ecoregion. ICES advice, book 6.

[CR33] Janßen H, Augustin C, Hinrichsen HH, Kube S (2013). Impact of secondary hard substrate on the distribution and abundance of *Aurelia aurita* in the western Baltic Sea. Mar Poll Bull.

[CR34] Ki JS, Hwang DS, Lee JS (2010). Simultaneous detection of *Aurelia* and *Chrysaora* scyphozoan jellyfish on a DNA microarray. J Mar Biol Ass UK.

[CR35] Lee PL, Dawson MN, Neill SP, Robins PE, Houghton JD, Doyle TK, Hays GC (2013). Identification of genetically and oceanographically distinct blooms of jellyfish. J Roy Soc Int.

[CR36] Leewis R, Waardenburg H (1991). Environmental impact of shipwrecks in the North Sea: I. Positive effects: epifauna of North Sea shipwrecks. Wat Sci Tech.

[CR37] Lengkeek W, Coolen JWP, Gittenberger A, Schrieken N (2013). Ecological relevance of shipwrecks in the North Sea. Ned Faun Meded.

[CR38] Lengkeek W, Didderen K, Dorenbosch M, Bouma S, Waardenburg HW (2013b) Biodiversiteit van kun-stmatige substraten. Een inventarisatie van 10 scheepswrakken op het NCP. Rapport 13-226. Tech. rep., Bureau Waardenburg, Culemborg

[CR39] Lindeyer F, Gittenberger A (2011). Ascidians in the succession of marine fouling communities. Aquat Invasions.

[CR40] Lucas CH (2001). Reproduction and life history strategies of the common jellyfish, *Aurelia aurita*, in relation to its ambient environment. Hydrobiologia.

[CR41] Lucas CH, Graham W, Widmer C (2012). Jellyfish life histories: role of Polyps in forming and maintaining Scyphomedusa populations. Adv Mar Biol.

[CR42] Ludwig W, Strunk O, Westram R, Richter L, Meier H, Buchner A, Lai T, Steppi S, Jobb G, Förster W (2004). ARB: a software environment for sequence data. Nucl Acids Res.

[CR43] Luttikhuizen PC, Stift M, Kuperus P, Van Tienderen PH (2007). Genetic diversity in diploid vs. tetraploid *Rorippa amphibia* (Brassicaceae). Mol Ecol.

[CR44] Makabe R, Furukawa R, Takao M, Si Uye (2014). Marine artificial structures as amplifiers of *Aurelia aurita* sl blooms: a case study of a newly installed floating pier. J Oceanogr.

[CR45] Meirmans PG, Van Tienderen PH (2004). GENOTYPE and GENODIVE: two programs for the analysis of genetic diversity of asexual organisms. Mol Ecol Notes.

[CR46] Merck T (1989) Untersuchungen zur ¨ okologischen Nische von *Chrysaora hysoscella*. Jahresb Biol Anst Helgol 53–54

[CR47] Miller B, Von der Heyden S, Gibbons M (2012). Significant population genetic structuring of the holo-planktic scyphozoan *Pelagia noctiluca* in the Atlantic Ocean. Afr J Mar Sci.

[CR48] Mills C (2001). Jellyfish blooms: are populations increasing globally in response to changing ocean conditions?. Hydrobiologia.

[CR49] Otto L, Zimmerman JTF, Furnes GK, Mork M, Saetre R, Becker G (1990). Review of the physical oceanography of the North Sea. Neth J Sea Res.

[CR50] Paradis E (2010). pegas: an R package for population genetics with an integrated–modular approach. Bioinformatics.

[CR51] Peijnenburg KT, Goetze E (2013). High evolutionary potential of marine zooplankton. Ecol Evol.

[CR52] Philippart CJM, Van Bleijswijk JDL, Kromkamp JC, Zuur AF, Herman PMJ (2014). Reproductive phenology of coastal marine bivalves in a seasonal environment. J Plankton Res.

[CR53] Pitt K (2000). Life history and settlement preferences of the edible jellyfish *Catostylus mosaicus* (Scyphozoa: Rhizostomeae). Mar Biol.

[CR54] Purcell J (2012). Jellyfish and ctenophore blooms coincide with human proliferations and environmental perturbations. Ann Rev Mar Sci.

[CR55] Purcell JE, Hoover RA, Schwarck NT (2009). Interannual variation of strobilation by the scyphozoan *Aurelia labiata* in relation to polyp density, temperature, salinity, and light conditions in situ. Mar Ecol Prog Ser.

[CR56] Quast C, Pruesse E, Yilmaz P, Gerken J, Schweer T, Yarza P, Peplies J, Glöckner FO (2013). The SILVA ribosomal RNA gene database project: improved data processing and web-based tools. Nucl Acids Res.

[CR57] Ramšak A, Stopar K, Malej A (2012). Comparative phylogeography of meroplanktonic species, *Aurelia* spp. and *Rhizostoma pulmo* (Cnidaria: Scyphozoa) in European Seas. Hydrobiologia.

[CR58] Richardson AJ, Bakun A, Hays GC, Gibbons MJ (2009). The jellyfish joyride: causes, consequences and management responses to a more gelatinous future. Trends Ecol Evol.

[CR59] Russell FS (1970). The Medusae of the British Isles II. Pelagic Scyphozoa, with a supplement to the first volume of Hydromedusae.

[CR60] Schiariti A, Christiansen E, Morandini AC, Da Silveira FL, Giberto DA, Mianzan HW (2012). Reproductive biology of *Lychnorhiza lucerna* (Cnidaria: Scyphozoa: Rhizostomeae): individual traits related to sexual reproduction. Mar Ecol Res.

[CR61] Schrieken N, Gittenberger A, Coolen JWP, Lengkeek W (2013). Marine fauna of hard substrata of the Cleaver Bank and Dogger Bank. Ned Faun Meded.

[CR62] Stopar K, Ramšak A, Trontelj P, Malej A (2010). Lack of genetic structure in the jellyfish *Pelagia noctiluca* (Cnidaria: Scyphozoa: Semaeostomeae) across European seas. Mol Phylogenet Evol.

[CR63] Straehler-Pohl I, Jarms G (2010). Identification key for young ephyrae: a first step for early detection of jellyfish blooms. Hydrobiologia.

[CR64] Straehler-Pohl I, Widmer CL, Morandini AC (2011). Characterizations of juvenile stages of some se-maeostome Scyphozoa (Cnidaria), with recognition of a new family (Phacellophoridae). Zootaxa.

[CR65] Thiel H (1962). Untersuchungen über die Strobilisation von *Aurelia aurita* Lam. an einer Population der Kieler Förde. Kieler Meeresforsch.

[CR66] Thiel M (1966). Untersuchungen über die Herkunft, das Auftreten, das Wachstum und die Fortpflanzung von *Rhizostoma octopus* L. Ag. im Elbmündungsgebiet. Abh Verhandl Naturwissens Ver Hamburg NF.

[CR67] Thomsen PF, Kielgast J, Iversen LL, Møller PR, Rasmussen M, Willerslev E (2012). Detection of a diverse marine fish fauna using environmental DNA from seawater samples. PLoS ONE.

[CR68] Toke D (2011). The UK offshore wind power programme: a sea-change in UK energy policy?. Energy Policy.

[CR69] Turrell W (1992). New hypotheses concerning the circulation of the northern North Sea and its relation to North Sea fish stock recruitment. ICES J Mar Sci.

[CR70] van der Baan SM (1980) The seasonal occurrence of Scyphomedusa in surface waters near the ‘Texel’ lightvessel. Intern verslag NIOZ 1980-8

[CR71] van der Molen J, van Beek J, Augustine S, Vansteenbrugge L, van Walraven L, Langenberg V, van der Veer HW, Hostens K, Pitois S, Robbens J (2015). Modelling survival and connectivity of *Mnemiopsis leidyi* in the south-western North Sea and Scheldt estuaries. Ocean Sci.

[CR72] van der Veer HW (1985). Impact of coelenterate predation on larval plaice *Pleuronectes platessa* and flounder *Platichthys flesus* stock in the western Wadden Sea. Mar Ecol Prog Ser.

[CR73] van Moorsel G (1993) Monitoring kunstriffen 1992. Tech. rep., Bureau Waardenburg, Culemborg

[CR74] van Moorsel G, Waardenburg H (1992) De fauna op wrakken in de Noordzee in 1990. Tech. rep., Bureau Waardenburg, Culemborg

[CR75] van Moorsel G, Waardenburg H, van der Horst J (1991) Het leven op en rond scheepswrakken en andere harde substraten in de Noordzee (1986 T/M 1990) een synthese. Tech. rep., Bureau Waardenburg, Culemborg

[CR76] van Walraven L, Langenberg VT, Dapper R, Witte J, Zuur A, van der Veer HW (2015). Long-term patterns in 50 years of scyphomedusae catches in the western Dutch Wadden Sea in relation to climate change and eutrophication. J Plankton Res.

[CR77] Vanagt TJ, Faasse M (2014) Development of hard substratum fauna in the Princess Amalia Wind Farm. Monitoring six years after construction. eCOAST Report 2013009. Tech. rep., eCoast

[CR78] Waardenburg H (1987) De fauna op een aantal scheepswrakken in de Noordzee in 1987. Tech. rep., Bureau Waardenburg, Culemborg

