# Supplementary figures and images for: Where are the polyps? Molecular identification, distribution and population differentiation of Aurelia aurita jellyfish polyps in the southern North Sea area
Source: Mar Biol. 2016 Jul 18;163:172. doi: 10.1007/s00227-016-2945-4 (PMC4949292; doi:10.1007/s00227-016-2945-4)

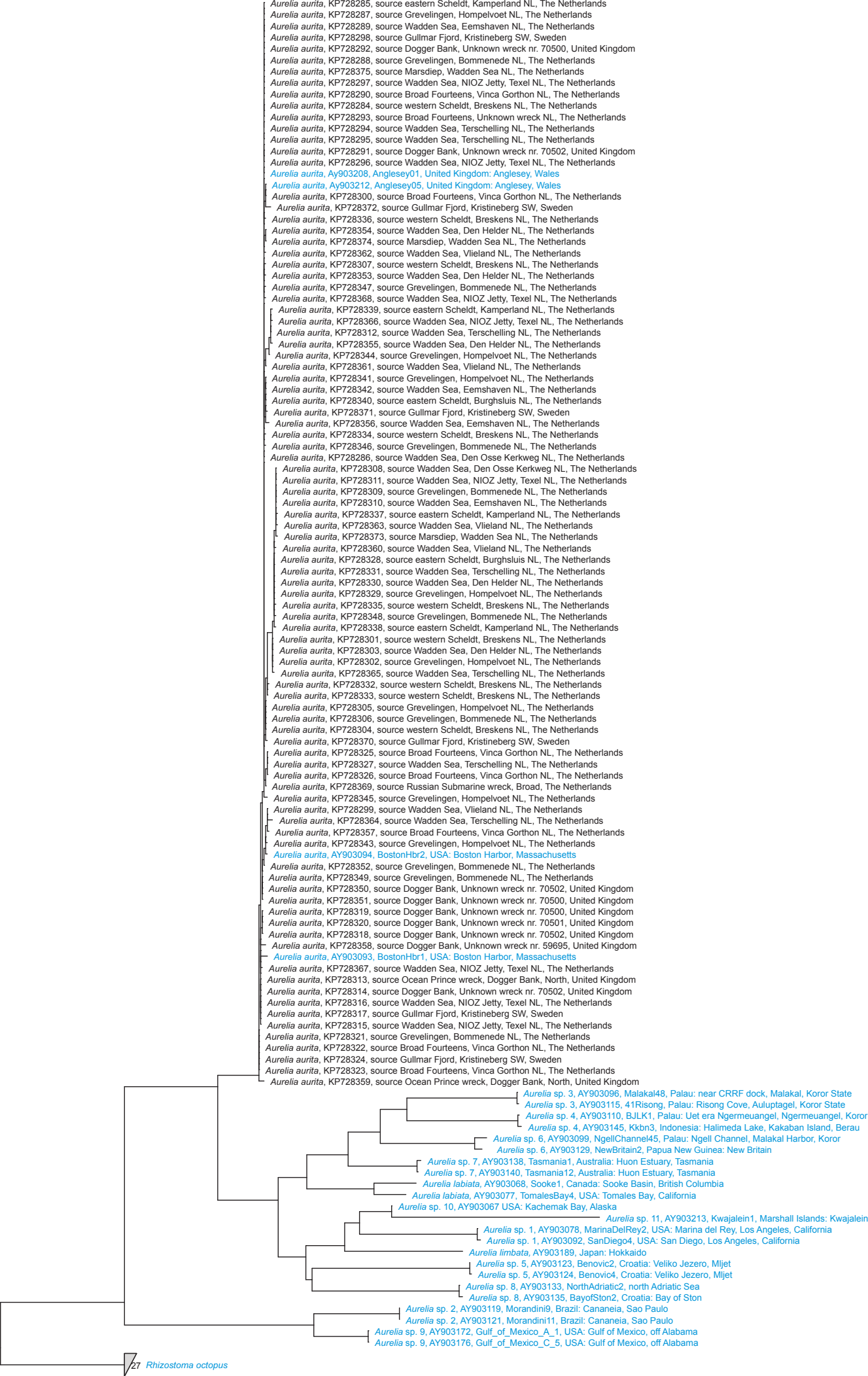

Supplement: Supplementary file 1 — Supplementary material 1 (PDF 44 kb) [file 227_2016_2945_MOESM1_ESM.pdf]
